# Correlates and Predictors of Increasing Waist Circumference in Patients with Type 2 Diabetes Mellitus: A Cross-Sectional Study

**DOI:** 10.1155/2014/318569

**Published:** 2014-09-15

**Authors:** Victor Mogre, Robert Abedandi, Zenabankara S. Salifu

**Affiliations:** ^1^Department of Human Biology, School of Medicine and Health Sciences, University for Development Studies, Tamale, Ghana; ^2^Department of Allied Health Sciences, School of Medicine and Health Sciences, University for Development Studies, Tamale, Ghana

## Abstract

Type 2 diabetes mellitus (type 2 DM) has become a disease of public health concern worldwide. Obesity and elevated blood pressure have been shown to be comorbidities of type 2 DM. In this cross-sectional study in Tamale, Ghana, we determined the prevalence of abdominal obesity among type 2 DM patients. Furthermore, we examined the demographic, clinical, and anthropometric predictors of increasing waist circumference in this population. Three hundred type 2 DM patients attending the outpatient diabetes clinic of the Tamale Teaching Hospital, Ghana, were recruited for the study. Waist circumference (WC) and hip circumferences were measured appropriately. Systolic blood pressure (SBP) and diastolic blood pressure (DBP) and fasting plasma glucose (FPG) were taken from the personal health record files of patients. Demographic data were obtained. Pearson correlation and multiple linear regression models were employed to identify predictors of increasing WC. The prevalence of abdominal obesity was 77.0% and was significantly higher in women than in men. A positive correlation was observed between waist-to-hip ratio (WHR) and WC (*r* = 0.56, *P* < 0.001), female gender (*r* = 0.73, *P* < 0.001), and age (*r* = 0.20, *P* < 0.001). A high prevalence of abdominal obesity was observed. Predictors of increasing WC were gender, age, FPG, and WHR.

## 1. Background

Diabetes especially diabetes mellitus (DM) has become a disease of concern for both developed and developing countries [1, 2]. In adults, the prevalence of DM worldwide was estimated to be 8.3% in 2011 and is expected to rise to 9.9% by the year 2030 [3] largely due to the global obesity epidemic and other factors [4]. A DM prevalence of 8% has been reported in Europe and the US [5, 6]. In sub-Saharan Africa the prevalence of diabetes has not been well established [7]. Reported prevalence varies widely (Benin 3%; Mauritania 6%; Cameroon 6.1%; Congo 7.1%; Zimbabwe 10.2%; Democratic Republic of Congo 14.5%) [8]. Type 2 diabetes mellitus (type 2 DM) has been shown to be the most common form of diabetes in sub-Saharan Africa [9, 10] constituting 90–95% [11]. At least 6% of adults in Ghana are affected with type 2 DM [12].

Type 2 DM has been shown to be associated with obesity [12]. Abdominal obesity/central obesity measured by either waist circumference (WC) or waist-to-hip ratio (WHR) has been shown to be strongly associated with diabetes and other chronic diseases than general obesity measured by BMI [13–15]. It is established that abdominal obesity is associated with decreased glucose tolerance, alterations in glucose insulin homeostasis, reduced metabolic clearance of insulin, and decreased insulin-stimulated glucose disposal [13]. Some 31% of adults in Tamale, Ghana, are centrally obese and this has been related to advanced age, low physical activity, female gender, urban environment, high income, distorted perception of weight status, and tertiary education [12, 16–21].

With contrasting prevalence, severe complications, and public health significance, a study on the prevalence of abdominal obesity and its predictors in type 2 DM patients are remarkably scarce. Understanding the burden of abdominal obesity and its associated factors is pertinent in guiding diagnosis, management, and prevention of DM in sub-Saharan Africa.

The objective of this study was to determine the prevalence of abdominal obesity (measured by WC) in a sample of type 2 DM patients. Furthermore, we examined the demographic, clinical, and anthropometric determinants of increasing waist circumference in this same sample.

## 2. Materials and Methods

### 2.1. Participants

Three hundred (300) previously diagnosed diabetes patients attending the diabetes clinic of the Tamale Teaching Hospital in Tamale, Ghana, were recruited for this cross-sectional study in 2013. As described elsewhere [21], all previously diagnosed diabetes patients that sought for care from the Hospital's diabetes clinic, during the study period, were eligible to participate in the study. Three hundred and twenty-one participants were approached; 300 of them consented to the study, yielding a response rate of 93.5%. Informed consent was obtained from all the study participants. The study was approved by the Ethics Committee of the University for Development Studies, School of Medicine and Health Sciences, Ghana.

### 2.2. Inclusion Criteria

Participants aged ≥ 30 years, who have clinical determined type 2 DM based on the WHO criteria [22], and who have duration of diabetes of at least 1 year were qualified to participate in the study.

### 2.3. Exclusion Criteria

Participants aged < 30 years and pregnant and lactating mothers were excluded from the study. Also participants with a history of heart failure, type 1 DM, myocardial infarction, acromegaly, hypothyroidism, hypogonadism, and any other chronic diseases, patients on prolonged steroid use, and those who were on active drug treatment for obesity at the time of admission were excluded from the study.

### 2.4. Anthropometric Parameters

Anthropometric measurements of waist and hip circumferences were taken. Waist circumference (WC) was measured midway between the inferior angle of the ribs and the suprailiac crest [23]. Hip circumference was measured as the maximal circumference over the buttocks in centimetres. Both measurements were measured to the nearest 1 cm using a nonstretchable fibre-glass measuring tape (Butterfly, China). During both measurements, participants stood in an upright position, with arms relaxed at the side, feet evenly spread apart, and body weight evenly distributed in accordance with the WHO expert consultation report on waist circumference and waist-to-hip ratio [23]. Abdominal obesity was determined as a waist circumference >102 cm in men and >88 cm in women according to the World Health Organization cut-off points and risk of metabolic complications for waist circumference [23].

### 2.5. Clinical Parameters

Clinical variables such as systolic blood pressure (SBP) and diastolic blood pressure (DBP) and fasting plasma glucose (FPG) values were taken from the personal health record files of the diabetic patients. Elevated blood pressure denoted a mean BP ≥ 140/90 mmHg and/or documented antihypertensive treatment [24]. Impaired fasting glycaemia (IFG) was defined as FPG ≥ 6.1 mmol l^−1^ [22]. Hyperglycaemia was determined by a FPG ≥ 7.0 [22]. IFG and hyperglycaemia were combined to denote uncontrolled diabetes. Sociodemographic data such as gender, age, and duration of diabetes were also obtained from the patients.

### 2.6. Statistical Analysis

All data were entered into Microsoft Excel 2007 and analysed using GraphPad Prism version 5 (GraphPad software, San Diego, California, USA, http://www.graphpad.com) and the PASW statistics 18 for Windows. Data are expressed as mean and SD. Continuous data were analyzed using Student's *t*-test whilst categorical variables were analyzed using Fisher's exact test using GraphPad Prism version 5. Pearson correlation coefficient was used to test the relationship among the various parameters. A Forward:LR method of multiple linear regression models was used to determine the predictors of WC. All correlation and multiple linear regression analyses were done using PASW statistics 18 for Windows. In all statistical tests, a value of *P* < 0.05 was considered significant.

## 3. Results

Presented in Table 1 are the general characteristics of the study population.

Mean waist circumference was found to be 95.99 cm and significantly higher in women than in men. The prevalence of abdominal obesity was found to be 77.0% and significantly higher in women than in men (*P* < 0.001). Average systolic blood pressure (SBP) and diastolic blood pressure (DBP) of the participants were 122.8 ± 16.16 mmHg and 84.5 ± 13.83 mmHg. Men had higher mean SBP (*P* = 0.015) and DBP (*P* = 0.001) than women. Over 70% of both men and women were found to have uncontrolled diabetes. Among the participants with uncontrolled diabetes, 29.9% (*n* = 69) had impaired fasting glycaemia and 70.1% (*n* = 162) had hyperglycaemia. Significantly (*P* = 0.008), the mean age of the women (57.22 ± 12.32 years) in this study was higher than men (52.83 ± 10.85 years).

The mean duration of diabetes among the participants was found to be 5.23 ± 5.00 years in which men had a higher mean duration than women.

As seen in Table 2, there were significant positive correlations between WHR and WC, gender, and age. Significantly, WC correlated positively with WHR (*r* = 0.56, *P* < 0.01), age (*r* = 0.50, *P* < 0.01), and gender (*r* = 0.36, *P* < 0.01).

The multiple linear regression models in Table 3 show the relative contribution of predictor variables of WC. Explaining 14% of the variance in WC in Step 1, gender was a significant predictor of WC. In Step 2, gender was no longer a significant predictor of WC, but WHR (*b* = 0.43, *P* < 0.01), age (*b* = 0.40, *P* < 0.01), and FPGL (*b* = 0.14, *P* < 0.01) were significant predictors of WC. The model explained 48% of the variance in predicting WC. DD did not significantly predict WC.

## 4. Discussion

This cross-sectional study from Tamale, Ghana, among type 2 DM patients attending an outpatient clinic shows a high prevalence of central obesity and a relatively low prevalence of elevated blood pressure. Parameters that significantly predicted increasing waist circumference were gender, age, FPG, WHR, and WC.

Evidently, the prevalence of abdominal obesity was found to be 77.0%. This is higher than the central obesity prevalence of 31.2% reported among an apparently healthy population of civil servants in Tamale, Ghana [18]. Our findings however concur with the central obesity prevalence of 75% in a hospital-based study of diabetes patients in Kumasi, Ghana [12], and 68.1% among diabetes patients in Southern India [25]. This is not surprising since obesity has been shown to be a significant risk factor for diabetes especially in type 2 DM [13, 14].

The prevalence of elevated BP was found to be 19.0% in this diabetic population. This is lower than the 63.0% reported among diabetes patients in Kumasi, Ghana [12], 54.2% among diabetes patients in Benin City, Nigeria [26], 66.4% in a Cameroonian type 2 diabetic population [27], and 50% of the diabetes patients in Kenya [28]. The differences could be due to variations in definitions of hypertension, population characteristics, and ethnic variations [29].

In a univariate analysis, females were severalfold at risk of developing abdominal obesity. This was confirmed by our correlation and multiple linear regression models in which the female gender strongly predicted WHR and WC in a positive direction. Several studies have reported findings that are in agreement with the association between the female gender and obesity among diabetics [30–33]. A similar relationship has also been reported in cross-sectional studies among apparently healthy populations [18, 34–36].

In identifying factors that influenced WC, our correlation and multiple linear regression models revealed WHR, age, and FPG as independent predictors of WC in a positive direction. As reported in several studies, a positive relationship was found between age and WC making age a strong predictor of WC (*β* = 0.40, 95% CI = 0.40–0.64, *P* < 0.01) [12, 37–39].

In agreement with our findings, WC significantly predicted the levels of blood glucose in a cross-sectional study in a sample of 5,882 adults from the US 1999–2004 National Health and Nutrition Examination Survey [40]. In a population-based survey of the prevalence of diabetes and correlates in an urban slum in a community in Nairobi, Kenya, Ayah et al. found that persons with diabetes were twice as likely to have an elevated WC (OR 2.3; 95% CI 1.2–4.6%) and WHR (OR 2.1; 95% 1.1–3.9%) [41]. Increased intra-abdominal adipose tissue is the most clinically relevant type of body fat that is associated with metabolic complications and adverse health effects including hyperinsulinemia and type 2 DM [42–44]. This indicates the negative influence of increasing WC on blood glucose levels. In the management of diabetes to maintain blood glucose levels, emphasis should be placed on regularly measuring and monitoring abdominal and central adiposity using a simple and inexpensive measure like WC [45].

Our findings should be considered in light of the following limitations. This was a cross-sectional study design that could not establish causality. FPG and blood pressure values were obtained secondarily from the personal health files of the diabetes patients. Although all care was taken to record the values to minimize errors, misreporting might have occurred. In addition, the sample size was not well distributed in terms of gender. Women were significantly higher than men; this affects the representativeness of the sample.

## 5. Conclusion

A high prevalence of central obesity was found. Predictors of increasing WHR and WC were the female gender, age, and FPG. A relatively low prevalence of elevated blood pressure was found.

## Figures and Tables

**Table 1:** Anthropometric and clinical measurements of the study population stratified by gender.

Variable	Total (*n* = 300)	Men (*n* = 69)	Women (*n* = 231)	*P* value
WC (cm)	95.99 ± 15.61	85.17 ± 13.93	99.22 ± 14.63	<0.001
Abdominal obesity				
Yes	231 (77.0%)	18 (26.1%)	213 (92.2%)	<0.001
No	69 (23.0%)	51 (73.9%)	18 (7.8%)	
WHR	0.94 ± 0.20	0.71 ± 0.06	1.00 ± 0.18	<0.001
SBP (mmHg)	122.8 ± 16.16	127 ± 12.04	121.6 ± 17.02	0.015
DBP (mmHg)	84.5 ± 13.83	88.26 ± 11.75	83.38 ± 14.23	0.001
Elevated BP				
Yes	57 (19.0%)	21 (30.4%)	36 (15.6%)	0.008
No	243 (81.0%)	48 (69.6%)	195 (84.4%)	
Age (years)	56.21 ± 12.12	52.83 ± 10.85	57.22 ± 12.32	0.008
≤40 years	33 (11.0%)	12 (17.4%)	21 (9.1%)	0.077
>40 years	267 (89.0%)	57 (82.6%)	210 (90.9%)	
FPG (Mean ± SD)	7.94 ± 2.81	8.07 ± 2.76	7.9 ± 2.83	0.653
Uncontrolled diabetes (%)	**231 (77.0%) **	**51 (73.9%) **	**180 (77.9%)**	**0.516**
IFG (%)	69/231 (29.9%)	18/51 (35.3%)	51/180 (28.3%)	0.387
Hyperglycaemia (%)	162/231 (70.1%)	33 (47.8%)	129/180 (71.7%)	
Duration (years)	5.23 ± 5.00	6.37 ± 4.9	4.88 ± 4.97	0.030
≤5 years	198 (66.0%)	39 (56.5%)	159 (68.8%)	0.062
>5 years	102 (34.0%)	30 (43.5%)	72 (31.2%)	

IFG: impaired fasting glycaemia.

**Table 2:** Pearson correlation coefficient of the study parameters (*n* = 300).

Variable	WHR	WC	AG	FPG	DD
Gender (GE)	***0.73∗∗***	**0.36∗∗**	0.15∗∗	−0.01	−0.16∗∗
WHR (WHR)		***0.56∗∗***	0.21∗∗	0.01	0.06
WC (WC)			**0.50∗∗**	0.05	−0.01
Age (AG)				−0.33∗∗	0.01
FPG (FPG)					−0.02

^**^Correlation is significant at the 0.01 level (2-tailed). WHR: waist-to-hip ratio; WC: waist circumference; AG: Age; FPG: fasting plasma glucose; and DD: diabetes duration.

**Table 3:** Multiple linear regression predicting waist circumference (*n* = 300).

Variable	*B* (SE)	*β*	95% CI
Step 1			
(Constant)	85.17 (1.74)		81.75–88.60
Female gender	14.04 (1.99)	0.38∗∗	10.14–17.95
Step 2			
(Constant)	36.56 (8.94)		18.98–54.14
Male versus female	1.86 (1.99)	0.05	−2.05–5.77
WHR	33.59 (4.24)	0.43∗∗	25.25–41.93
Duration	−0.020 (0.14)	−0.01	−0.29–0.25
Age (years)	0.52 (0.06)	0.40∗∗	0.40–0.64
FPG (m/mol)	0.75 (0.24)	0.14∗	0.27–1.23

*R* ^2^ for Step  1 = 0.14, Step  2 = 0.48 (*P* < 0.001). ∗∗*P* < 0.001, ∗*P* < 0.05. WHR: waist-to-hip ratio; WC: waist circumference; AG: age; FPG: fasting plasma glucose; and DD: diabetes duration.
